# Malaria Hotspots Drive Hypoendemic Transmission in the Chittagong Hill Districts of Bangladesh

**DOI:** 10.1371/journal.pone.0069713

**Published:** 2013-08-06

**Authors:** Sabeena Ahmed, Sean Galagan, Heather Scobie, Jacob Khyang, Chai Shwai Prue, Wasif Ali Khan, Malathi Ram, Mohammad Shafiul Alam, M. Zahirul Haq, Jasmin Akter, Gregory Glass, Douglas E. Norris, Myaing Myaing Nyunt, Timothy Shields, David J. Sullivan, David A. Sack

**Affiliations:** 1 Centre for Population, Urbanization and Climate Change, International Centre for Diarrhoeal Disease Research, Bangladesh, Dhaka, Bangladesh; 2 Johns Hopkins Malaria Research Institute, Johns Hopkins Bloomberg School of Public Health, Baltimore, Maryland, United States of America; Mahidol University, Thailand

## Abstract

**Background:**

Malaria is endemic in 13 of 64 districts of Bangladesh, representing a population at risk of about 27 million people. The highest rates of malaria in Bangladesh occur in the Chittagong Hill Districts, and *Plasmodium falciparum* (predominately chloroquine resistant) is the most prevalent species.

**Methods:**

The objective of this research was to describe the epidemiology of symptomatic *P. falciparum* malaria in an area of Bangladesh following the introduction of a national malaria control program. We carried out surveillance for symptomatic malaria due to *P. falciparum* in two demographically defined unions of the Chittagong Hill Districts in Bangladesh, bordering western Myanmar, between October 2009 and May 2012. The association between sociodemographics and temporal and climate factors with symptomatic *P. falciparum* infection over two years of surveillance data was assessed. Risk factors for infection were determined using a multivariate regression model.

**Results:**

472 cases of symptomatic *P. falciparum* malaria cases were identified among 23,372 residents during the study period. Greater than 85% of cases occurred during the rainy season from May to October, and cases were highly clustered geographically within these two unions with more than 80% of infections occurring in areas that contain approximately one-third of the total population. Risk factors statistically associated with infection in a multivariate logistic regression model were living in the areas of high incidence, young age, and having an occupation including jhum cultivation and/or daily labor. Use of long lasting insecticide-treated bed nets was high (89.3%), but its use was not associated with decreased incidence of infection.

**Conclusion:**

Here we show that *P. falciparum* malaria continues to be hypoendemic in the Chittagong Hill Districts of Bangladesh, is highly seasonal, and is much more common in certain geographically limited hot spots and among certain occupations.

## Background

Malaria is endemic in 106 countries globally and caused an estimated 216 million cases and between 655,000 and 1.2 million deaths in 2010 [1], [2]. Hypoendemic malaria transmission, defined as transmission where less than 10% of 2–9 year olds are positive for malaria parasites, currently threatens over one billion people in South and Southeast Asia [3]. In hypoendemic settings, transmission is unstable and tends to occur in seasonal outbreaks based on climatic factors [4]–[7]. Furthermore, a higher proportion of malaria cases are symptomatic, and severe malaria tends to occur at any age due to lack of meaningful immunity from decreased exposure to malaria parasites, in contrast to high transmission areas of sub-Saharan Africa [6], [8]. Understanding the patterns of transmission and risk factors for malaria infection in these hypoendemic areas is essential for developing effective malaria control strategies, especially as programs aim toward elimination.

In Bangladesh, malaria is endemic in 13 of 64 districts, representing a population at risk of 26.9 million people [9], [10]. Over the previous decade the national malaria control program in Bangladesh has reported more than 50,000 cases per year and between 100 to 500 deaths per year [9]; however, it is likely that the burden of malaria is underestimated. Malaria in Bangladesh occurs seasonally with the majority of malaria infections in the rainy season from May to October [11], [12], and transmission is variable across endemic districts with the highest incidence in the Chittagong Hill Districts (CHD) of Southeast Bangladesh [12], [13]. Most infections are caused by *Plasmodium falciparum*, although between 5–20% of infections are reported to be caused by *P. vivax* [11], [14]. *P. ovale* and *P. malariae* infections have been reported, but are extremely rare [15], [16].

The CHD is an ethnically diverse region of Southeast Bangladesh encompassing the three districts (Bandarban, Khagrachari, and Rangamati) with the highest malaria burden in Bangladesh [12], [13]. The majority of its residents are from one of twelve different ethnic tribal groups with a minority of non-tribal Bengali, Bangladesh's major ethnic group [17]. These inhabitants live predominantly in forested, rural areas, and daily labor and agriculture are the most common occupations. This includes the practice of jhum cultivation, a form of shifting cultivation where ethnic tribal groups grow crops on remote, steep hillsides [18], [19]. The remote, forested ecozone of the CHD, contiguous with Myanmar, as well as the occupational and behavioural risk factors of its inhabitants may explain the higher malaria incidence in the region.

Starting in 2007, the Government of Bangladesh received funding from the Global Fund to fight Malaria, AIDS and Tuberculosis to implement a national malaria control program. The program works with various non-governmental organizations but most importantly BRAC, which provides long-lasting insecticide treated bed nets at the village level at no cost and supports treating old nets with deltamethrine in the insecticide-treated nets program [20]. The nets distributed include Net Protect (Bestnet, UK), Olyset (Sumitomo Co. Ltd., Japan), and Permanet (Verstergaard Fradsen, Switzerland) and are believed to be effective for 3–5 years [21].

In 2009, the International Centre for Diarrhoeal Disease Research, Bangladesh (icddr, b) in collaboration with the Johns Hopkins Malaria Research Institute initiated a malaria surveillance system in the CHD [10]. Two unions (subunit of sub-district) were selected for the study area: Kuhalong and Rajbila in the Bandarban Sadar sub-district. These unions were selected because they are typical of areas of high risk in the CHD, the high prevalence observed during the initial survey [13], and its accessibility. The purpose of this surveillance system was to study the epidemiology of malaria in the region, validate diagnostic methods, and better understand the entomology of malaria vectors in Southeast Asia [10], [14], [22], [23].

This paper investigates the epidemiology of symptomatic *P. falciparum* malaria infection over two years of prospective malaria surveillance in Kuhalong and Rajbila unions. The objectives of the study were to determine the incidence of infection over the study period, investigate seasonal variation of malaria transmission in this region, map spatial variation of malaria incidence across the study area, and identify risk factors for symptomatic *P. falciparum* infection in a hypoendemic setting of Southeast Bangladesh.

## Methods

### Study population

Demographic surveillance captured a population of 23,372 individuals and 4,782 households from October 2009 to May 2012. This over-inflates the true population at any one time due to deaths (n = 206) and because in-migration and out-migration were common during the study period. Households were divided into 24 study clusters (12 in each union) for programmatic purposes comprising approximately 1,000 people per cluster [10]. Clusters were defined following an initial census to create working units for the surveillance activities.

### Study design and data collection

A population census was conducted in March to April 2009 to collect global positioning system (GPS) locations of households and to count the number of people in the study area. Prospective passive surveillance for malaria infection was performed by local surveillance workers from October 2009 in Kuhalong union and May 2010 in Rajbila union through April 2012. Individuals reporting fever or other malaria symptoms were tested at the village level for malaria infection by microscopic examination of a blood smear and rapid diagnostic test using FalciVax test strip, which has been validated as a detection method for both *P. falciparum* (97.6% sensitive, 95.8% specific) and *P. vivax* (76.5% sensitive, 100% specific) in this study area [14]. Microscopy was performed by standard protocols. At the start of the surveillance period, 44 positive and negative slides were examined by the study microscopist and an experienced microscopist from an independent laboratory not associated with the study. With the exception of minor discrepancies on quantification, there was 100% agreement between the readings. Individuals testing positive for any type of malaria infection by either test were treated with artemether-lumefantrine and then retested using the same methods as well as PCR analysis of dried blood on filter paper on days 2, 7, and 28. Individuals were clinically evaluated to ensure the infection had resolved.

Surveillance workers collected sociodemographic information at both the individual and household levels. The initial demographic survey was conducted from October 2009 to February 2010 in Kuhalong and April to August 2010 in Rajbila. Surveillance workers also periodically collected follow-up demographic information to ascertain births, deaths, in-migration, out-migration, and pregnancies [10].

### Statistical Analysis

This analysis was restricted to symptomatic *P. falciparum* malaria infections detected by passive (symptomatic) surveillance from May 2010 to April 2012 and documented by either rapid diagnostic test or by microscopic examination of a blood smear. Individuals with malaria infection detected through passive surveillance prior to May 2010 (n = 8), through active screening for asymptomatic cases (n = 70), or due to *P. vivax* infection (n = 24), were not included in this analysis. Furthermore, positive tests occurring in the same individual within 28 days of a previous positive test were not counted as a new infection and removed from the analysis (n = 1). Infections were categorized as either a high transmission season infection (May to October) or a low transmission season infection (November to April). It should be noted that some individuals had multiple infections and their demographic information was counted more than once in the analysis. However, this was an uncommon event (n = 42, 0.2% of study population) and did not significantly alter the results.

Demographic variables of interest in this analysis included individual characteristics such as age, sex, location (by union), ethnicity, education level, occupation, and bed net use (reported as the bed net use the previous night before the survey was administered). Household characteristics included distance from the house to the forest or pond and the presence of animals nearby (at the house). Responses were coded into binary and categorical variables for analysis. Binary variables included sex (male vs. female), study union (Rajbila vs. Kuhalong), and household animal ownership (yes or no). Age was divided into infants <6 months, children 6–59 months, children 5–14 years old, and adolescents and adults ≥15 years old. Ethnicities included non-tribal Bengali, Marma, Tanchangya, Tripura, Chakma, Khyang, and other less common tribal groups categorized into a single ‘other tribal’ ethnic group (Bawm, Mro, and Rakhaine). The level of education was defined as low (0–2 years), medium (3–5 years), and high (≥6 years). Occupations of interest included daily labor, agriculture, jhum cultivation, and other occupations (mostly housewives, students, and unemployed). Due to the nature of the surveys, individuals could report jhum cultivation and another profession, but due to the hypothesized risk associated with jhum cultivation these individuals were coded as jhum cultivation only. Finally, we investigated household distance to ponds (no pond, 0–50 meters, 50–100 meters, >100 meters) and the forest (0–25 meters, 25–50 meters, >50 meters).

Sociodemographic covariates were tabulated to describe the study population, and incidence rates of symptomatic *P. falciparum* infection were calculated across sociodemographic variables to identify risk factors for infection. Incidence rates were calculated by dividing the number of symptomatic *P. falciparum* infections over the study period by the total person-time at risk across all sociodemographic covariates of interest. Person-time at risk (in months) was calculated for each individual by subtracting time lost due to death or out-migration and due to person-time not in the study area before birth or in-migration during the study period from 24 months (the total study period). It should be noted that the dates of death, in-migration and out-migration were only accurate to the date at which the surveillance worker detected the event (follow-up surveillance occurred every 3–4 months). Incidence rates were reported as the number of infections per month per 1,000 individuals. Incidence rates were calculated for both individual and household characteristics in both the high and the low transmission seasons. Statistically significant differences in the incidence rates within a sociodemographic group were detected using Poisson regression. All statistical analyses were conducted in Stata (version 12.1; Statacorps, College Station, USA).

The effect of age on symptomatic *P. falciparum* incidence was further delineated by plotting a histogram of incidence rates by age. Individuals were divided into 5-year age groups with 0–5 year olds split into infants <6 months old and children 6–59 months old, and the oldest individuals consolidated into a single ≥75 years old age group. The monthly symptomatic *P. falciparum* incidence rate per 1,000 was calculated and plotted for each group.

The maximum and minimum altitude and symptomatic *P. falciparum* incidence rates, including the number of cases, the total population and the total person-time (in months) at risk, are reported for each study cluster. The coefficient of variation was calculated for all incidence rates as well as within each union to determine the variability in symptomatic *P. falciparum* incidence in the study area. The correlation between maximum and minimum altitude and symptomatic incidence rates was explored by calculating correlation coefficients.

Univariate and multivariate logistic regression analyses were conducted to determine the true sociodemographic predictors of malaria infection. All demographic and household factors were used in this analysis. Furthermore, a new covariate representing geography was included to control for spatial variation in malaria incidence across study clusters. Individuals were defined as living in a high, medium, or low-incidence cluster based on the calculated incidence rates of in each study cluster and each category included 8 of the 24 total study clusters. Univariate logistic regression modelling the odds of malaria infection compared to a reference group was conducted for each sociodemographic factor. For multivariate logistic regression, the true sociodemographic predictors of malaria infection were selected by forward and backward selections with a likelihood-ratio test (p = 0.1). Covariates with a likelihood-ratio test where p>0.1 were not included in the final model. Furthermore, the multivariate model was validated using goodness-of-fit testing (Hosmer-Lemeshow and Pearson's chi^2^ tests) and by collinearity checks using variance inflation factors. It should be noted that this analysis did not account for differences in person-time between individuals as logistic regression was used instead of survival analysis or Poisson regression.

While symptomatic *P. vivax* infections were not considered in the bulk of this analysis, the incidence of symptomatic *P. vivax* infection was stratified by age and transmission season and calculated by dividing the number of *P. vivax* infections that were observed during the study period by the total person-months at risk (# cases/month per 1,000). Statistically significant differences in the incidence rates between different ages were detected using Poisson regression.

### Climatic factors and malaria infections

Bandarban city weather data provided by the Ministry of Agriculture, Soil Resources Development Institute was used to compare monthly symptomatic *P. falciparum* infection levels with average daily weather metrics over the study period. The average values of daily humidity (%), daily high and low temperatures (°C), and daily distribution of rainfall (mm) per month were overlaid on a histogram of symptomatic *P. falciparum* infections per month from May 2010 to April 2012. The distribution of rainfall is a transformation of average rainfall that takes into account the number of days that it rained in a month (distribution of rainfall = average daily rainfall per month * the number of rainy days in the month) [24]. The correlation between the number of monthly symptomatic *P. falciparum* cases and humidity, temperature, or rainfall data collected in Bandarban city was evaluated.

### Mapping incidence rates and households

A map showing the study area, study clusters, and household locations was generated to show spatial variation in symptomatic *P. falciparum* incidence rates. Households were mapped using GPS locations from the original census, and study clusters were generated based on distances from households. Study clusters were categorized and labelled based on calculated monthly incidence rates with clusters labelled 0.05–0.25 (n = 7 clusters), 0.26–0.50 (n = 6 clusters), 0.51–1.09 (n = 6 clusters) or 1.10–3.46 (n = 5 clusters) cases per 1,000 individuals per month. Households were labelled by the ethnicity of the household. This map was created in ArcGIS (version 10.0; ESRI, Redlands USA).

### Ethical considerations

The study protocol and consent forms were approved by the icddr, b Ethical Review Committee and the Johns Hopkins Bloomberg School of Health Institutional Review Board. Written informed consent was obtained from participants prior to enrolment into the study or in case of minors/children from their guardians. A copy of the consent form was provided to the head of the household once informed consent was obtained. The study was conducted in accordance with the principles of research ethics stated in the Declaration of Helsinki and the local and international regulatory guidelines.

## Results

### Description of the study population

Demographic surveillance collected information from 23,372 individuals from October 2009 to April 2012. The characteristics of the study population are described in Table 1. The population was relatively young, with 37.7% of the population less than 15 years old. Ethnic tribal groups represented the majority (79.4%) of the study population, and the Marma (60.1%) were the largest group. The most common occupations for adults ≥ 15 years old were agriculture (18.4%), jhum cultivation (17.5%), and daily labor (13.7%), and most adults had little education (59.0% with 0–2 years). The reported bed net use was high close to 90%. Approximately 70%–80% of households were located close to animal dwelling and/or forested areas.

**Table 1:** Description of the study population.

Demographic and household factors		N (%)
Union	Rajbila	10,498 (44.9)
	Kuhalong	12,874 (55.1)
Sex	Males	11,456 (49.0)
	Females	11,916 (51.0)
Age	<6 months	988 (4.2)
	6–59 months	2,621 (11.2)
	5–14 years	5,200 (22.3)
	≥15 years	14,563 (62.3)
Ethnicity	Bengali	4,821 (20.6)
	Total tribal	18,551 (79.4)
	Marma	14,035 (60.1)
	Tanchangya	2,085 (8.9)
	Khyang	1,134 (4.9)
	Chakma	802 (3.4)
	Tripura	391 (1.7)
	Other tribal	104 (0.4)
Education level (age ≥15 years)	0–2 years	8,598 (59.0)
	3–5 years	2,668 (18.3)
	≥6 years	3,297 (22.6)
Occupation (age ≥15 years)	Agricultural	4,609 (31.7)
	Day labor	1,996 (13.7)
	Jhum cultivation	2,544 (17.5)
	Other	5,414 (37.2)
Bed net use	Yes	18,869 (89.3)
	No	2,255 (10.7)
Distance from house to pond	0–50 meters	4,304 (18.4)
	50–100 meters	1,897 (8.1)
	>100 meters	1,012 (4.3)
	No pond	16,141 (69.1)
Distance from house to forest	0–25 meters	7,869 (33.7)
	25–50 meters	9,187 (39.1)
	>50 meters	6,316 (27.0)
Own animals	No	4,285 (18.3)
	Yes	19,087 (81.7)
Total		23,372

### Temporal relationship between malaria cases and climatic factors

**Figure 1:**
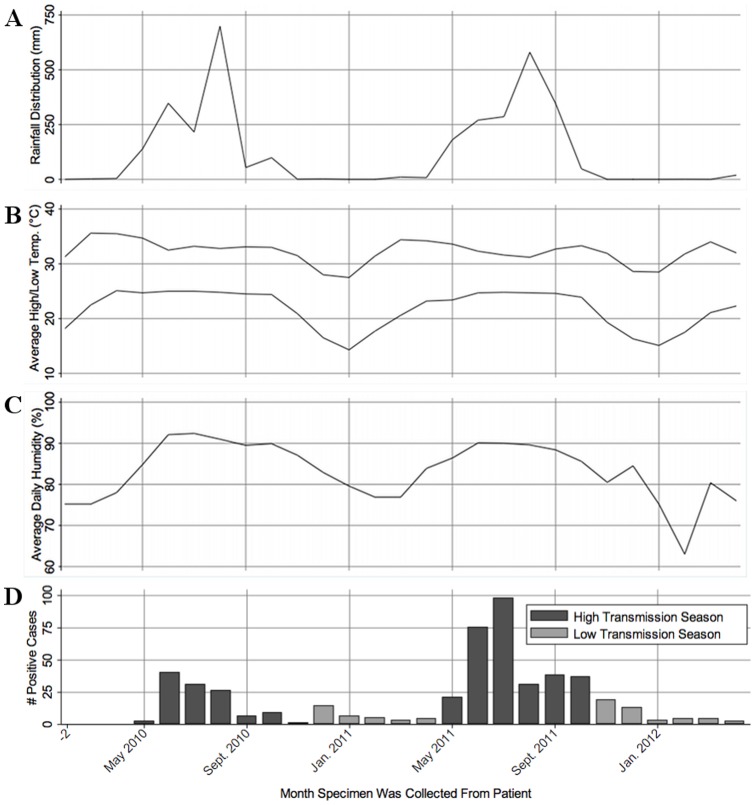
Positive malaria cases and climatic factors per month, May 2010 to April 2012. Symptomatic *P. falciparum* infection numbers were correlated with (**A**) rainfall distribution (R^2^ = 0.252; p = 0.007), (**B**) average daily minimum temperature (R^2^ = 0.203; p = 0.016), and (**C**) average daily humidity (R^2^ = 0.261; p = 0.006). Case numbers were not associated with (**D**) the average daily maximum temperature (R^2^ = 0.002; p = 0.820). Rainfall distribution is defined by the average daily rainfall per month (in mm) multiplied by the number of rainy days in that month.

From May 2010 to April 2012 there were a total of 472 symptomatic *P. falciparum* infections, with 405 infections (85.8%) in the high transmission season from May to October, and 67 infections (14.2%) in the low transmission season from November to April (Figure 1). Cases peaked in June and July in both 2010 and 2011 and case numbers in 2011 were higher than 2010. A significant correlation was found between the number of symptomatic *P. falciparum* infection and climatic factors. *P. falciparum* infections increased during the rainy season (R^2^ = 0.252; p = 0.007) (Figure 1A), when the average daily low temperature increased (R^2^ = 0.203; p = 0.016) (Figure 1B), and when the average daily humidity was above 80% (R^2^ = 0.261; p = 0.006) (Figure 1C). The number of cases was not significantly correlated to the average daily high temperature (R^2^ = 0.002; p = 0.820) (Figure 1B).

### Spatial variation in symptomatic *P. falciparum* incidence

**Figure 2:**
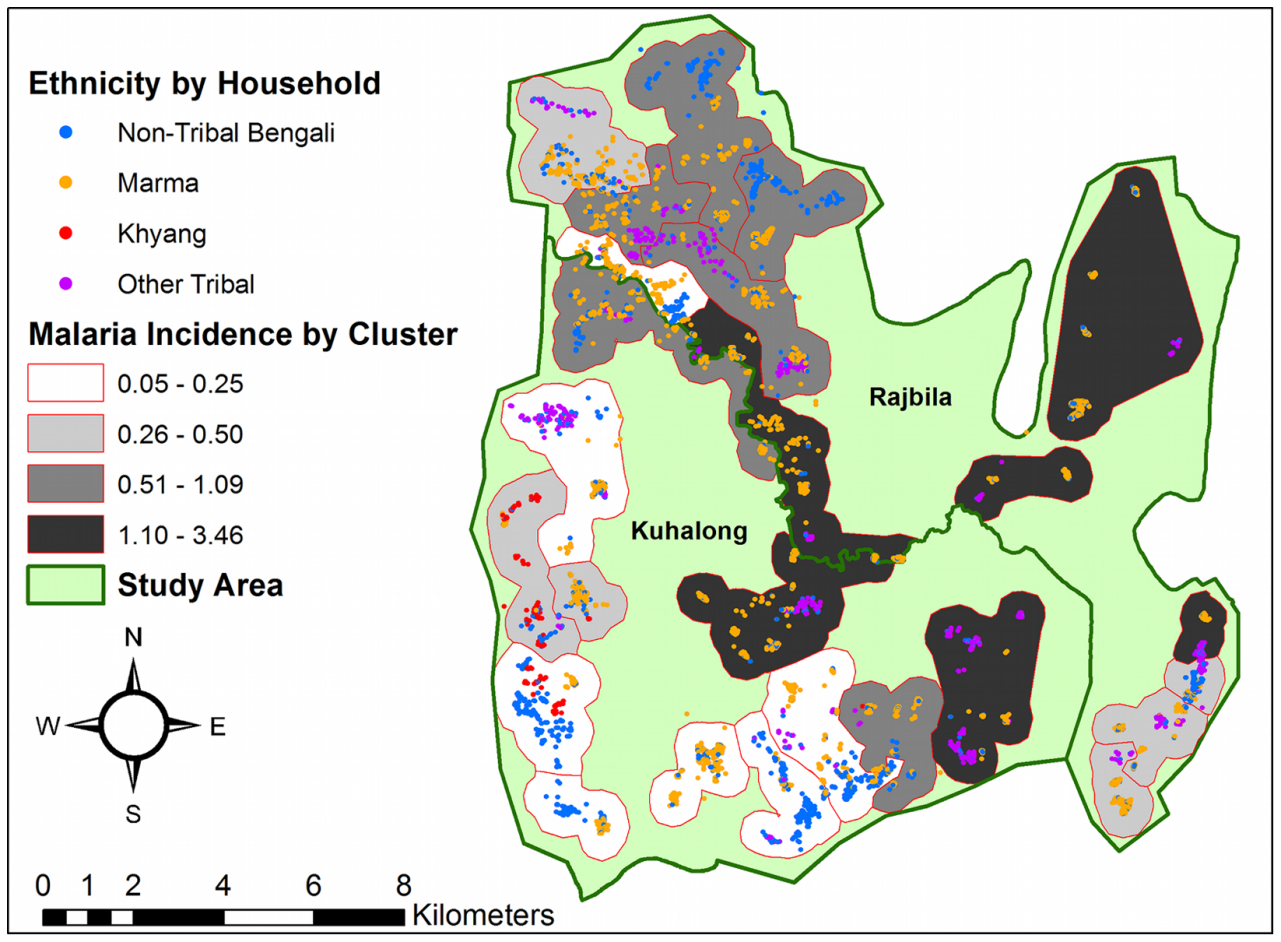
Monthly malaria incidence by surveillance cluster, May 2010 to April 2012. Malaria incidence is reported as the number of symptomatic *P. falciparum* infections per 1,000 individuals per month. The non-tribal Bengalis and the Khyang tribe predominately lived in low incidence areas. The malaria incidence for the Marmas varied by location of household amongst the clusters.

Symptomatic *P. falciparum* incidence rates were calculated for all study clusters from May 2010, to April 2012, and labelled on a map of the study area with household locations labelled by ethnic group (Figure 2). The highest incidence rates (1.10 to 3.46 cases per 1,000 per month) occurred in the eastern part of both study unions and in the center of the study area on the border between the two unions, which are almost exclusively inhabited by Marma and other tribal groups. The lowest incidence rates (0.05 to 0.50 cases per 1,000 per month) occurred in the south-western clusters in Kuhalong, which are inhabited by non-tribal Bengalis, the Khyang, and a small number of tribal households. Intermediate incidence (0.50–1.09 cases per 1,000 per month) was seen in the north-western region of Kuhalong and especially Rajbila where a mixture of tribal groups and Bengalis live. Of the 472 cases detected over the study period, 362 (76.7%) cases occurred in the eight highest incidence clusters (n = 7,549), 84 (17.8%) cases occurred in eight intermediate incidence clusters (n = 7,337), and 26 (5.5%) cases occurred in eight lowest incidence clusters (n = 8,488).

### Symptomatic *P. falciparum* incidence by sociodemographic factors

**Figure 3:**
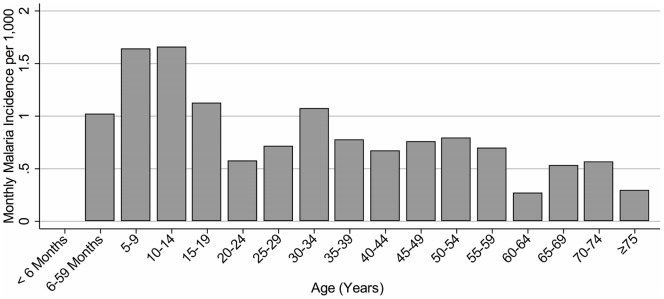
Monthly malaria incidence by age, May 2010 to April 2012. Malaria incidence is reported as the number of symptomatic *P. falciparum* infections per 1,000 individuals per month. Children age 5 to 14 years had the highest incidence rates of any 5-year age groups.

Symptomatic *P. falciparum* incidence rates (number of cases per 1,000 per month) were calculated across five-year age groups (Figure 3). Children ≤5 years old were split into infants <6 months and children 6–59 months and all adults ≥75 years were combined into a single category. There were no cases in infants <6 months old and incidence rates increased through childhood, peaking at 1.64 cases and 1.66 cases per 1,000 per month in children 5–9 and 10–14 years old, respectively. After childhood, incidence rates declined, with the lowest incidence rates (<0.6 cases per 1,000 per month) occurring in the elderly ≥60 years old.

Symptomatic *P. falciparum* incidence rates were also calculated across household and demographic factors in both the high and low transmission seasons (Tables 2–3). Overall, the incidence of symptomatic *P. falciparum* infection was 1.62 cases per 1,000 per month in the high transmission season, a six-fold increase from the low transmission season (0.27 cases per 1,000 per month) (Table 2). In the high transmission season, there was statistically significant variation in incidence rates across study unions, between males and females (marginally), across ethnic groups, across different occupations, and between those who did and did not use bed nets (Table 2). The groups with the highest incidence rates were children 5–14 years old (2.75 cases per 1,000 per month), individuals who reported not using a bed net the previous night (2.66 cases per 1,000 per month), individuals who conducted jhum cultivation (2.42 cases per 1,000 per month), people living in Rajbila (2.05 cases per 1,000 per month), and ethnic tribal groups besides the Khyang (ranging from 1.74 to 20.70 cases per 1,000 per month). In the low transmission season, the only variation that occurred in symptomatic *P. falciparum* incidence rates was between different tribal groups and different age groups.

**Table 2:** Monthly malaria incidence rates by transmission season and sociodemographic factors.

		High transmission season			Low transmission season		
	Total	Incidence rate per			Incidence rate per		
Household factors	Population	Cases	1,000/month	p =	Cases	1,000/month	p =
***Union***							
Rajbila	10,498	234	2.05	**<0.001**	34	0.30	0.413
Kuhalong	12,874	171	1.26		33	0.24	
***Sex***							
Males	11,456	217	1.78	0.053	35	0.29	0.570
Females	11,916	188	1.47		32	0.25	
***Age***							
<6 months	985	0	0	**<0.001**	0	0	**<0.001**
6–59 months	2,621	43	1.48		16	0.55	
5–14 years	5,200	149	2.75		23	0.42	
≥15 years	14,563	213	1.34		28	0.18	
***Ethnicity***							
Bengali	4,821	33	0.64	**<0.001**	6	0.12	**<0.001**
Total tribal	18,551	372	1.88		61	0.31	
Marma	14,035	275	1.84		34	0.23	
Tanchangya	2,085	39	1.74		9	0.40	
Khyang	1,134	3	0.24		5	0.40	
Chakma	802	15	1.74		7	0.81	
Tripura	391	20	5.42		4	1.08	
Other tribal	104	20	20.70		2	2.07	
***Education level (age ≥15 years)***							
0–2 years	8,598	136	1.40	0.604	16	0.17	0.338
3–5 years	2,668	38	1.32		8	0.28	
≥6 years	3,297	39	1.18		4	0.12	
***Occupation (age ≥15 years)***							
Agricultural	4,609	48	0.93	**<0.001**	11	0.21	0.643
Day labor	1,996	42	1.91		5	0.23	
Jhum cultivation	2,544	71	2.42		5	0.17	
Other	5,414	52	0.92		7	0.12	
***Bed net use***							
Yes	18,869	306	1.48	**<0.001**	54	0.26	0.611
No	2,255	58	2.66		7	0.32	
Total	23,372	405	1.62		67	0.27	

**Table 3:** Monthly malaria incidence rates by transmission season and household factors.

		High transmission season			Low transmission season		
			Incidence rate per			Incidence rate per	
Household factors	Total Population	Cases	1,000/month	p =	Cases	1,000/month	p =
***Own animals***							
Yes	19,087	337	1.65	0.407	126	0.26	0.923
No	4,285	68	1.48		30	0.27	
***Distance from house to pond***							
0–50 meters	4,312	31	0.68	**<0.001**	7	0.15	**0.015**
50–100 meters	1,897	13	0.64		1	0.05	
>100 meters	1,022	13	1.19		5	0.46	
No pond	16,141	345	1.99		54	0.31	
***Distance from house to forest***							
0–25 meters	7,869	183	2.16	**<0.001**	34	0.40	**0.013**
25–50 meters	9,187	126	1.29		22	0.23	
>50 meters	6,316	96	1.43		11	0.16	
Total	23,372	405	1.62		67	0.27	

Variation in the incidence of symptomatic *P. falciparum* infection between individuals living at different distances to ponds and the forest in both the high and low transmission seasons was observed (Table 3). Individuals living closer to the forest had higher incidence rates than those living further away, and individuals living within 100 meters of ponds had lower incidence rates compared to those living further from ponds or not near ponds at all. There was no statistically significant difference in the incidence of infection between those who owned animals and those who did not in either transmission season.

The maximum and minimum altitude, total population, number positive symptomatic *P. falciparum* cases, total person-time (in months) at risk, and symptomatic *P. falciparum* incidence rates in each study cluster are shown in Table 4. Incidence rates ranged from 0.05 to 3.46 cases per 1,000 per month with a coefficient of variation of 1.16. There was greater variability in incidence rates in Kuhalong with a coefficient of variation of 1.41 compared to 0.97 in Rajbila. The altitudes of households in each study cluster were relatively similar across study clusters with a range of 9 to 48 meters minimum to 44 to 147 meters maximum. Symptomatic *P. falciparum* incidence rates were strongly associated with maximum (R^2^ = 0.742, p<0.001), but not minimum (R^2^ = 0.180; p = 0.022) altitude.

**Table 4:** Symptomatic *P. falciparum* incidence rates and other selected characteristics of study clusters.

Union	Cluster	Minumum altitude (m)	Maximum altitude (m)	Total Population	Cases	Person time at risk (months)	Incidence rate per1,000/month
Rajbila	1	24	57	964	9	20,717	0.43
	2	26	67	755	17	16,557	1.03
	3	44	99	853	21	19,209	1.09
	4	48	101	1,078	16	23,856	0.67
	5	46	89	748	10	16,176	0.62
	6	32	55	946	1	20,833	0.05
	7	43	125	880	44	18,688	2.35
	8	32	141	735	53	15,324	3.46
	9	45	147	1,165	75	24,904	3.01
	10	27	45	806	5	17,543	0.29
	11	28	63	839	9	18,341	0.49
	12	31	56	729	8	16,153	0.50
	Total	24	57	10,498	268	228,301	1.17
	Intercluster coefficient of variation						0.97
Kuhalong	1	17	57	933	8	20,897	0.38
	2	20	44	1,147	8	24,703	0.32
	3	29	72	899	3	18,723	0.16
	4	34	71	899	16	18,969	0.84
	5	29	106	1,300	77	28,197	2.73
	6	15	51	1,102	6	23,883	0.25
	7	16	52	1,364	3	27,720	0.11
	8	21	61	1,016	21	20,975	1.00
	9	31	105	843	54	16,970	3.18
	10	12	47	1,301	2	27,497	0.07
	11	13	49	845	4	16,589	0.24
	12	9	77	1,225	2	25,506	0.08
	Total	17	57	12,874	204	270,629	0.75
	Intercluster coefficient of variation						1.41
**Total**				23,372	472	498,930	0.95
	Intercluster coefficient of variation						1.16

### Unadjusted and adjusted logistic regression model to predict symptomatic *P. falciparum* infection by sociodemographic factors

The odds of symptomatic *P. falciparum* infection depending on different sociodemographic characteristics were modelled using univariate and multivariate logistic regression analysis (Table 5). The following characteristics were associated with increased odds of infection compared to a referent group: living in Rajbila compared to living in Kuhalong (OR = 1.6), children 5–14 years old compared to adults ≥15 years old (OR = 2.1), ethnic tribal groups (except the Khyang) compared to non-tribal Bengalis (OR≥2.8), jhum cultivation compared to other occupations (OR = 1.7), and living near the 0–25 meters from the forest compared to ≥50 meters from the forest (OR = 1.5). Bed net use was associated with a decrease in the odds of infection compared to those who did not (OR = 0.6).

**Table 5:** Unadjusted and adjusted odds of high transmission season malaria infection by sociodemographic characteristics.

		Unadjusted (N = 23,372)		Adjusted (N = 22,549)	
Demographic and household factors		OR (95% CI)	p =	OR (95% CI)	p =
Union	Rajbila	1.6 (1.4, 2.0)	**0.001**	Not Selected^b^	
	Kuhalong	1.0			
Geography	Low-incidence clusters	1.0		1.0	
	Medium-incidence clusters	4.3 (2.6, 7.0)	**<0.001**	4.2 (2.5, 7.0)	**<0.001**
	High-incidence clusters	18.3 (11.6 28.7)	**<0.001**	13.9 (8.7, 22.2)	**<0.001**
Sex	Males	1.2 (1.0, 1.5)	0.064	Not Selected^b^	
	Females	1.0			
Age	<6 months	−a		−a	
	6–59 months	1.2 (0.9, 1.7)	0.277	1.3 (0.8, 2.0)	0.245
	5–14 years	2.1 (1.7, 2.6)	**<0.001**	2.9 (2.0, 4.1)	**<0.001**
	≥15 years	1.0		1.0	
Ethnicity	Bengali	1.0		1.0	
	Marma	2.9 (2.0, 4.2)	**<0.001**	1.3 (0.8, 1.9)	0.259
	Tanchangya	2.8 (1.7, 4.4)	**<0.001**	1.2 (0.7, 2.0)	0.425
	Khyang	0.4 (0.1, 1.3)	0.114	0.2 (0.1, 1.0)	0.055
	Chakma	2.8 (1.5, 5.1)	**0.001**	1.0 (0.5, 1.8)	0.921
	Tripura	7.8 (4.4, 13.8)	**<0.001**	1.4 (0.8, 2.5)	0.276
	Other tribal	34.5 (19.0, 62.7)	**<0.001**	6.3 (3.3, 12.1)	**<0.001**
Education level	0–2 years	1.0		1.0	
	3–5 years	0.9 (0.7, 1.2)	0.680	1.1 (0.8, 1.7)	0.470
	≥6 years	0.6 (0.5, 0.9)	**0.004**	1.1 (0.7, 1.5)	0.776
Occupation	Agricultural	0.6 (0.4, 0.8)	**0.001**	0.9 (0.6, 1.4)	0.637
	Day labor	1.2 (0.9, 1.7)	0.301	2.0 (1.4, 3.0)	**0.001**
	Jhum cultivation	1.7 (1.3, 2.2)	**<0.001**	1.5 (1.0, 2.1)	**0.030**
	Other	1.0		1.0	
Bed net use	Yes	0.6 (0.5, 0.8)	**0.001**	Not Selected^b^	
	No	1.0			
Distance from house to pond	0–50 meters	0.3 (0.2, 0.5)	**<0.001**	0.6 (0.3, 0.8)	**0.003**
	50–100 meters	0.3 (0.2, 0.5)	**<0.001**	0.5 (0.3, 0.9)	**0.013**
	>100 meters	0.6 (0.3, 1.0)	0.065	0.6 (0.3, 1.0)	**0.046**
	No pond	1.0		1.0	
Distance from house to forest	0–25 meters	1.5 (1.2, 2.0)	**0.001**	1.1 (0.8, 1.4)	0.516
	25–50 meters	0.9 (0.7, 1.2)	0.445	0.8 (0.6, 1.1)	0.159
	>50 meters	1.0		1.0	
Own Animals	Yes	1.1 (0.9, 1.4)	0.418	Not Selected^b^	
	No	1.0			
Age ≥15 years * years education	0–2 years	-	-	1.0	
	3–5 years	-	-	0.4 (0.2, 1.0)	0.059
	≥6 years	-	-	0.7 (0.4, 1.2)	0.248
Goodness-of-fit tests				Chi^2^ test	p =
Hosmer-Lemeshow		-	-	12.5 (8 df)	0.131
Pearson's chi^2^ test		-	-	1192.2 (1607 df)	1.000

OR =  odds ratio; 95% CI = 95% confidence interval.

a Could not be modelled due to lack of variability in the outcome (no infections).

b Not selected into final model after forward and backward stepwise selections by likelihood-ratio test (p>0.1).

After using forward and backward selections to generate a final multivariate model to identify the sociodemographic factors that are associated with symptomatic *P. falciparum* incidence, the following factors were not related to infection: Bed net use, gender, and whether or not the household owned animals (Table 5). After controlling for other factors, the strongest predictors of infection were age, occupation, and location. Those living in high-incidence and medium-incidence clusters had much higher odds of infection compared to those in low-incidence clusters (OR = 13.9 and 4.2 respectively). Children 5–14 years old were significantly more likely to be infected (OR = 2.9) as compared to adults ≥15 years old. Both jhum cultivation (OR = 1.5) and daily labor (OR = 2.0) significantly increased the odds of infection as compared to other occupations (students, the unemployed, housewives, and others). After controlling for other factors, ethnic tribal groups were no longer associated with increased odds of infection compared to non-tribal Bengalis. This occurred due to the inclusion of the variable representing spatial variation in malaria incidence across the study area. The only exception was tribal minorities (the Bawm, Mro, and Rakhaine) (OR = 6.3), which only represented 0.4% of the study area.

### The incidence of symptomatic *P. vivax* incidence by age and transmission season

There were a total of 24 symptomatic *P. vivax* infections observed from May 2010 to April 2012 for an overall incidence of 0.05 cases per 1,000 individuals per month (Table 6), There was little difference in incidence rates between transmission seasons, with 13 cases in the high transmission season (0.03 cases per 1,000 per month) and 11 cases in the low transmission season (0.02 case per 1,000 per month). There were no cases in infants <6 months old. Children 6–59 months and 5–14 years old had the highest incidence rates of *P. vivax* (0.07 and 0.08 cases per 1,000 per month compared to 0.03 cases per 1,000 per month in adults ≥15 years old), although this relationship was not statistically significant.

**Table 6:** Symptomatic *P. vivax* incidence rates by age and transmission season.

Demographic factors	Total population	*P. vivax* Cases	Incidence rate per 1,000/month	p =
***Age***				
<6 months	985	0	0	0.141
6–59 months	2,621	4	0.07	
5–14 years	5,200	9	0.08	
≥15 years	14,563	11	0.03	
***High transmission season***	23,372	13	0.03	
***Low transmission season***	23,372	11	0.02	
**Total**	23,372	24	0.05	

## Discussion

This study of the epidemiology of symptomatic *P. falciparum* infection confirms that malaria in this area of the CHD is typical of hypoendemic settings with highly seasonal patterns of transmission. Though rates of symptomatic malaria were higher in the younger age groups, age-specific differences are less pronounced in this area than in hyperendemic areas [8], [25].

Symptomatic malaria was highly clustered in this region, both in time and space. Since our study area is relatively small (179 sq. km) and appears to be fairly uniform in geographic characteristics, the major differences in rates were somewhat surprising. These geographic hotspots for malaria transmission, encompassing approximately a third of the total population, appear to account for a majority nearly 80% of all the symptomatic malaria infections in these two unions. This approximates the 20/80 rule delineated by Woolhouse, et al. which suggests that malaria, like many infections, has clustered high transmission reservoirs [26].

As expected, symptomatic malaria rates were highly seasonal as more than 85% of diagnosed cases occurred in the high transmission season from May to October. These incidence rates correlated with variations in rainfall, humidity, and low (but not high) temperatures. This correlation with climatic events is consistent with findings from other endemic areas [5], [27], but following these trends over several more years will be needed to determine if climatic trends are determining factors for the severity of the malaria season from year to year in this specific region.

We evaluated several risk factors that correlate with higher rates, including ethnicity, occupation, and non-use of bed nets. We did not observe any genetic basis for malaria risk across different ethnicities in this study. Though tribal groups do have higher rates of symptomatic infection than the non-tribal Bengali population, this difference appears to be related to geographic location rather than ethnicity as controlling for spatial variation in malaria incidence in our multivariate model dissipated any effect of ethnicity on malaria incidence. Certain occupations, including jhum cultivation and daily labor, are associated with higher rates of infection, as is residence close to the forest. The observed high incidence of symptomatic infection in jhum cultivators will be further explored in an additional manuscript.

Interestingly, use of bed nets did not appear to be protective in the multiple logistic regression model, suggesting the need to carefully study the impact of bed nets in malaria control in this region. It is possible that our binary classification of bed net use was overly simplistic and more in-depth evaluations of bed net use, such as evaluating the effect of the number of bed nets in the house or the methods of bed net use and maintenance, are needed to understand the effect of bed net use on malaria infection. This lack of association may also be due to the presence of exophilic *Anopheles* vectors that had been previously identified as important malaria vectors in Bangladesh [28], [29]. Recent studies have identified these vectors in this study area and while they made up less than 3% of mosquitoes trapped [22], [23], they may be more abundant outside of villages where traps were located in these studies. Given that many people in this area work in the forest at jhum sites, rubber plantations, and doing daily labor, it is conceivable that malaria transmission may be occurring by these forest mosquitoes, which cannot be not be controlled by the use of bed nets. However, it should be noted that the studies by Rosenberg and Maheswary [28], [29] were conducted in a different area of Bangladesh in a holoendemic setting and may not be representative in this setting.

Symptomatic *P. vivax* infections only accounted for 5% of total symptomatic malaria infections from May 2010 to April 2012 with a monthly incidence of 0.05 cases per 1,000 compared to 0.95 symptomatic *P. falciparum* cases per 1,000 per month. These results are consistent with other reports from Bangladesh [11] and this study area [14]. Similar to *P. falciparum* infections, the highest *P. vivax* incidence rates occur in children 6–59 months and 5–14 years old. It is likely that symptomatic *P. vivax* rates are under-estimated as lower sensitivity of FalciVax rapid diagnostic testing (76.9%) of *P. vivax* infections has been observed in this study area [14]. Therefore, further studies in this area are needed to identify the burden of *P. vivax* infection.

These results are consistent with findings from other studies in the CHD [30], [31]. Similarly to our study, one study from Rajasthali sub-district (Rangamati district) found that before controlling for other factors in a multivariate model, ethnic tribal groups had higher odds of infections as compared to non-tribal Bengalis [31]. However, in both studies, this effect was not significant after controlling for other factors. Furthermore, similarly to our study, living closer to the forest increased the odds of infection. However, there were some differences. Haque, et al. found that having three or more bed nets in a household was protective against malaria infection [31] whereas our study only looked at bed net use the previous night and found that after controlling for other factors there was no effect of bed net use on malaria infection. Our study is consistent with earlier findings from Khagrachari District of Bangladesh where individuals living closer to ponds had lower rates of malaria than those living further away [30].

This paper focuses on symptomatic malaria due to *P. falciparum,* but does not include in its analysis asymptomatic infections, which are also very common. These will be reported in a separate paper; however, it should be stated that in this hypoendemic area, asymptomatic infections are more than twice as common as symptomatic infections.

Some strengths and limitations of this study should be noted. The major strength of this study is the surveillance among all age groups for symptomatic malaria in a demographically defined population in this area of Bangladesh, near the border with Myanmar. Thus, this data appears typical of malaria in Southeast Asia, an area of intense interest because of the threat of artemisinin resistance and the large population at risk to malaria infection [32]. Combining the demographic surveillance with geographic, sociodemographic, and meteorological data, this dataset is able to identify potential risk factors in this area, which has not been studied in depth previously. Since this project was carried out during the introduction of an enhanced national malaria control program, information from this study can be used to improve the strategies of that program. A limitation is the relatively short duration of surveillance; a two-year period is not sufficient to understand variability in rates in relation to climate over the long term.

Future plans are to continue malaria surveillance in this area for many years to identify climatic and other factors that relate to malaria rates over a longer period, to further define potential risk factors, and to evaluate potential interventions for interrupting transmission. Since most malaria cases occur in certain hot spots, future studies will focus on these geographic areas and certain occupation groups to evaluate potential interventions targeting these groups.

## Conclusion

This study showed that symptomatic *P. falciparum* malaria in two unions of the CHD of south-eastern Bangladesh exhibits highly seasonal, hypoendemic transmission that predominantly occurs in geographical hotspots. More than 80% of infections occurred in approximately one-third of the population over a two-year study period. Furthermore, infections are clustered in time as more than 85% of infections occurred in the high transmission season from May to October. This analysis also identified specific sociodemographic groups who are at increased risk of infection, such as children and adolescents 5–14 years, jhum cultivators, and day laborers.

Having identified temporal, spatial, and sociodemographic clusters of symptomatic *P. falciparum* infection following the introduction of a national malaria control program, further studies are needed to understand the dynamics of malaria transmission in these groups. Given the low numbers of cases that occur in the low transmission season, in much of the study area, and in certain geographic groups, these groups are highly susceptible to interventions that may be able to interrupt transmission. As malaria control programs in hypoendemic areas begin to shift from control to elimination, identifying and targeting clusters of infection and developing strategies to interrupt transmission in low malaria transmission settings will be essential.
